# Osteoprotegerin (OPG) activates integrin, focal adhesion kinase (FAK), and Akt signaling in ovarian cancer cells to attenuate TRAIL-induced apoptosis

**DOI:** 10.1186/1757-2215-6-82

**Published:** 2013-11-23

**Authors:** Denis Lane, Isabelle Matte, Claude Laplante, Perrine Garde-Granger, Claudine Rancourt, Alain Piché

**Affiliations:** 1 Département de Microbiologie et Infectiologie, Université de Sherbrooke, 3001, 12ième Avenue Nord, Sherbrooke, Québec J1H 5 N4, Canada; 2 Département de Pathologie, Faculté de Médecine, Université de Sherbrooke, 3001, 12ième Avenue Nord, Sherbrooke J1H 5N4, Canada

**Keywords:** Osteoprotegerin (OPG), TRAIL, Ovarian carcinoma, Resistance, Akt, Integrin, FAK

## Abstract

**Background:**

Resistance to apoptosis is a major problem in ovarian cancer (OC) and correlates with poor prognosis. Osteoprotegerin (OPG) is a soluble secreted factor that acts as a decoy receptor for receptor activator of NF-κB ligand (RANKL) and tumor necrosis factor-related apoptosis-inducing ligand (TRAIL). OPG has been reported to attenuate TRAIL-induced apoptosis in a variety of cancer cells, including OC cells. OPG-mediated protection against TRAIL has been attributed to its decoy receptor function. However, OPG activates integrin/focal adhesion kinase (FAK) signaling in endothelial cells. In OC cells, activation of integrin/FAK signaling inhibits TRAIL-induced apoptosis. Based on these observations, we hypothesized that OPG could attenuate TRAIL-induced apoptosis in OC cells through integrin/FAK signaling.

**Methods:**

*In vitro* experiments including immunoblots, colony formation assays, and apoptosis measurements were used to assess the effect of OPG on TRAIL-induced apoptosis.

**Results:**

Exogenous OPG protected from TRAIL-induced apoptosis in a TRAIL binding-independent manner and OPG protection was αvβ3 and αvβ5 integrin/FAK signaling-dependent. Moreover, OPG-mediated activation of integrin/FAK signaling resulted in the activation of Akt. Inhibition of both integrin/FAK and Akt signaling significantly inhibited OPG-mediated attenuation of TRAIL-induced apoptosis. Although OPG also stimulated ERK1/2 phosphorylation, inhibition of ERK1/2 signaling did not significantly altered OPG protection.

**Conclusions:**

Our studies provide evidence, for the first time, that OPG can attenuate TRAIL-induced apoptosis in a TRAIL binding-independent manner through the activation of integrin/FAK/Akt signaling in OC cells.

## Introduction

Osteoprotogerin (OPG) is a secreted member of the TNF receptor superfamily that was originally characterized based on its ability to suppress osteoclast formation [1,2]. OPG binds to the receptor activator of NF-κB ligand (RANKL) and functions as a soluble decoy receptor for RANKL. In bone, OPG inhibits osteoclastogenesis by preventing RANKL from binding to its receptor RANK and, consequently promotes apoptosis of osteoclast [1]. OPG is critical for osteoclastogenesis and, therefore, homeostasis of bone remodeling and bone mass [3]. In addition to its role in bone metabolism, OPG has been implicated in mucosal immunity [4] and vascular systems. OPG is secreted by endothelial cells [5,6] and promotes both proliferation and migration of microvascular endothelial cells [7,8], and induces angiogenesis [8-10]. OPG can also serve as survival factor for endothelial cells [6,8]. Furthermore, OPG acts as a decoy receptor of TNF-related apoptosis-inducing ligand (TRAIL) and neutralizes its function [11,12]. TRAIL belongs to the TNF family of cytokines and has emerged as a promising anticancer agent because of its ability to selectively induce apoptosis in a broad host of tumor cells [13]. TRAIL binding to its receptors (TRAIL-R1 and TRAIL-R2) initiates the extrinsic pathway of apoptosis, resulting in recruitment of the adapter protein Fas-associated death domain (FADD) and procaspase-8 in the death inducing signaling complex (DISC). Caspase-8 can directly activate the effector caspases (caspase‒3, ‒6, ‒7) leading to the execution of apoptosis [14]. However, in ovarian cancer cells, the apoptotic signal must be further amplified by engaging the intrinsic (mitochondrial) pathway [15]. In this context, caspase-8 cleaves Bid to generate an active tBid, which in turn activates proapoptotic Bax or Bak proteins, and induces mitochondrial outer membrane permeabilization (MOMP). The mitochondria then release proapoptotic factors that promote effector caspases activation.

Several reports have shown that OPG is a survival factor that can block TRAIL-induced apoptosis in tumor cells. Human prostate cancer cells were shown to secrete OPG at concentrations sufficient to inhibit TRAIL-induced apoptosis *in vitro*[16,17]. Similarly, multiple myeloma cells were protected from TRAIL-induced apoptosis by OPG secreted from osteoblast-like cells and bone marrow stroma cells [18]. OPG produced by breast cancer cells enhances tumor cell survival *in vitro* and *in vivo* by inhibiting TRAIL-induced apoptosis [19-22]. The production of OPG in colorectal cancer cells and the addition of exogenous OPG to colorectal cancer cells both caused resistance to TRAIL-induced apoptosis [23]. Exogenous addition of OPG also mediates resistance to TRAIL-induced apoptosis in ovarian cancer cells [24].

Because OPG binds to TRAIL, OPG-mediated protection from TRAIL in various cancer cells has been assumed to be mainly related to its decoy function. However, the observations that OPG activates integrin/focal adhesion kinase (FAK)/ERK1/2 signaling in endothelial cells [7,8] to promote proliferation and migration suggest that OPG regulates cell function directly. Indeed, it was suggested that OPG-mediated proliferation and migration of endothelial cells occurs in a TRAIL-independent manner [7,25]. In ovarian cancer cells, activation of integrin/FAK and ERK1/2 signaling contribute to attenuate TRAIL-induced apoptosis [26,27]. Based on these observations, we hypothesize that OPG might attenuate TRAIL-induced apoptosis in a TRAIL binding-independent manner by activating survival signaling pathways in ovarian cancer cells. The purpose of this study was to investigate whether exogenous OPG can confer protection against TRAIL-induced apoptosis independent from its ability to act as a TRAIL decoy receptor.

## Results

### OPG attenuates TRAIL-induced apoptosis in a TRAIL binding-independent manner

**Figure 1:**
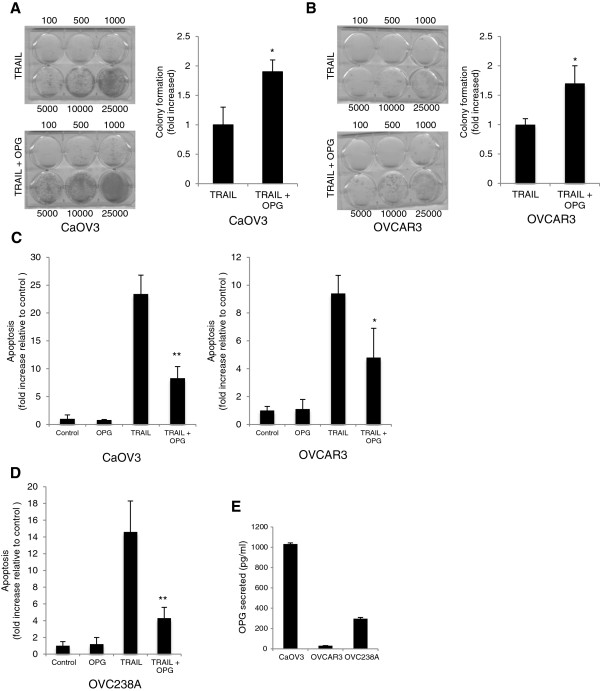
**OPG attenuates TRAIL-induced apoptosis in a TRAIL binding-independent manner.** CaOV3 cells **(A)** and OVCAR3 cells **(B)** were preincubated for 1 h with OPG (25 ng/ml), washed extensively to remove any OPG, and treated with TRAIL (50 ng/ml) for 48 h. The cells were then washed, seeded at different densities and incubated in fresh medium. Viable colonies were counted after 14 days and data were expressed at fold increase relative to control (untreated) cells. Results are from three independent experiments and express as mean fold increase ± SD. * *P* < 0.01 compared to control. CaOV3 and OVCAR3 cells **(C)** and primary tumor cells OVC238A **(D)** were preincubated for 1 h with OPG (25 ng/ml), washed and incubated with TRAIL (50 ng/ml) for 24 h, and apoptosis was assessed. Apoptosis is expressed as fold increase relative to control (untreated) cells with the mean of triplicates from three independent experiments ± SD. * *P* < 0.01; ** *P* < 0.001. **(E)** Concentration of OPG released in conditioned medium from CaOV3, OVCAR3 and OVC238A cells measured by ELISA.

To assess the hypothesis that OPG attenuates TRAIL-induced apoptosis in a TRAIL binding-independent manner, ovarian cancer cell lines CaOV3 and OVCAR3 were challenged with exogenous OPG for 1 h, washed extensively and incubated in medium containing TRAIL. OVCAR3 is an ovarian carcinoma cell line isolated from malignant ascites that is resistant to clinically relevant concentrations of cisplatin but remains sensitive to TRAIL-induced apoptosis. CaOV3 is also an ovarian carcinoma cell line isolated from a patient with advanced disease. The TRAIL signaling cascade has been well characterized in these cell lines [26-28]. The concentration of OPG was selected based on our previous study, which demonstrated that OPG, at a concentration of 25 ng/ml, significantly attenuates TRAIL-induced apoptosis [24]. OVCAR3 and CaOV3 cells were thus incubated with OPG for 1 h and cells were extensively washed to remove any OPG. Cells were then incubated in fresh medium containing TRAIL (50 ng/ml) for 48 h. Cell viability was assessed by clonogenic survival assays. Preincubation with OPG significantly increased the number of viable colonies in both CaOV3 (Figure 1A) and OVCAR3 (Figure 1B) cells when compared to cells that were not challenged with OPG before being treated with TRAIL (*P* < 0.01). In agreement with these findings, preincubation with OPG followed by its removal before cells were challenged with TRAIL attenuated TRAIL-induced apoptosis, as measured by oligosomal DNA fragmentation, in both CaOV3 and OVCAR3 cells (Figure 1C). To confirm the biological relevance these findings, primary OC tumor cells isolated from malignant ascites (OVC238A) were preincubated with OPG for 1 h, washed, and challenged with TRAIL. As shown in Figure 1D, OPG significantly attenuated TRAIL-induced apoptosis in these tumor cells (*P* < 0.001). To ensure that the amount of endogenous OPG secreted by CaOV3, OVCAR3 and OVC238A did not contribute to inhibit TRAIL-induced apoptosis, we measured the levels of OPG in conditioned medium from these cells. As shown in Figure 1E, the levels of OPG secreted in conditioned medium were below 1 ng/ml whereas the concentration of OPG required to provide TRAIL protection is ≥ 10 ng/ml in ovarian cancer cells [24]. All together, these data suggest that OPG may attenuate TRAIL-induced apoptosis independently from its decoy receptor action on TRAIL.

### OPG attenuates TRAIL-induced apoptosis through an integrin-dependent pathway

**Figure 2:**
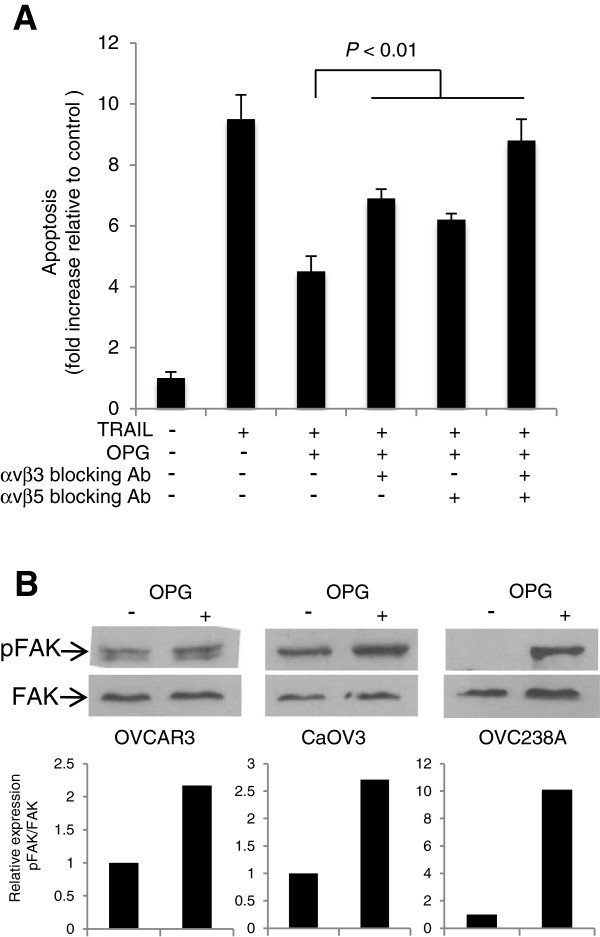
**Involvement of integrin/FAK signalling in OPG-mediated protection from TRAIL. (A)** CaOV3 cells were incubated with αvβ3 and αvβ5 integrin blocking antibodies (5 μg/ml) for 1 h. Cells were washed and incubated with OPG (25 ng/ml). After 1 h, cells were washed and TRAIL (50 ng/ml) was added for 24 h and apoptosis was assessed. Apoptosis is expressed as fold increase relative to control (untreated) cells with the mean of triplicates from three independent experiments ± SD. **(B)** CaOV3 cells were incubated with 25 ng/ml OPG for 1 h. Cells were lysed and subjected to immunoblotting to detect total and phosphorylated FAK. Densitometric quantification of phosphorylated FAK from three separate experiments normalized to total FAK was performed.

OPG-induced endothelial cell proliferation and migration was shown to be mediated by both αvβ3 and αvβ5 integrin suggesting that OPG may activate cell signaling [7]. Interestingly, we previously showed that signaling through αvβ5 integrin attenuated TRAIL-induced apoptosis in OC cells [26]. Because these data suggest that integrins might be involved in OPG-mediated inhibition of TRAIL-induced apoptosis in ovarian cancer cells, we examined the effect αvβ3 and αvβ5 blocking antibodies on OPG-mediated inhibition of TRAIL-induced apoptosis. CaOV3 cells, which express both αvβ3 and αvβ5 integrin [26], were incubated with anti-integrin blocking antibodies for 1 h followed by addition of OPG for 1 h. Cells were washed and TRAIL was added. As shown in Figure 2A, pre-incubation with αvβ3 or αvβ5 blocking antibodies significantly (*P* < 0.01) reduced the protective effect of OPG on TRAIL-induced apoptosis. The maximal reduction of OPG protection however was observed when both blocking antibodies were added together (Figure 2A).

The engagement of integrin to its ligand triggers a signaling cascade that leads to the activation of FAK, one of the earliest even downstream in integrin signaling [29]. Consistent with the role of integrin in OPG-mediated attenuation of TRAIL-induced apoptosis, we found that FAK was phosphorylated when OVCAR3 and CaOV3 cells were incubated with OPG while the levels of total FAK remained relatively stable (Figure 2B). We also observed a significant and stronger increase in the phosphorylation of FAK in primary OVC238A cells treated with OPG (Figure 2B). This could be related to the differential expression of integrins in ovarian cancer cell lines compared to primary ovarian cancer specimens [30]. Nonetheless, these data suggest that both αvβ3 and αvβ5 integrin signaling, which results in FAK activation, are involved in OPG-mediated attenuation of TRAIL-induced apoptosis.

### An Akt-dependent pathway mediates OPG-induced attenuation of TRAIL-induced apoptosis

**Figure 3:**
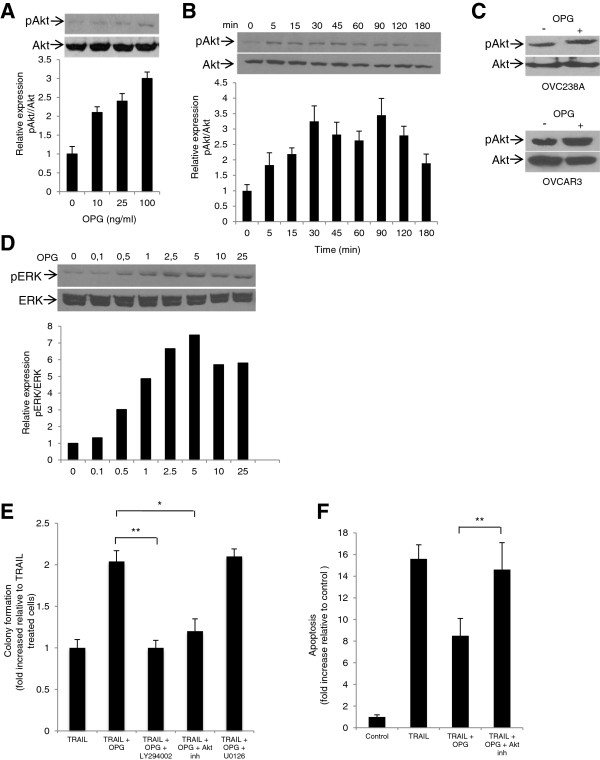
**OPG attenuates TRAIL-induced apoptosis in an Akt-dependent manner.** CaOV3 cells were treated with increasing concentrations (0–100 ng/ml) of OPG **(A)** or with 25 ng/ml OPG for various times (0–180 min) **(B)**. Cells were lysed, and the levels of total and phosphorylated Akt were determined by immunoblot. Densitometric quantification of phosphorylated Akt from three separate experiments normalized to total Akt was done. **(C)** OVCAR3 and OVC238A cells were treated with 25 ng/ml OPG and 60 min later, cells were lysed and immunoblot was performed to determine the levels of total and phosphorylated Akt. **(D)** CaOV3 cells were treated with increasing concentrations (0–25 ng/ml) of OPG and total and phosphorylated ERK1/2 were determined by immunoblot. The levels of phosphorylated ERK1/2 were determined by densitometric quantification. **(E)** CaOV3 cells were preincubated with LY294002 (5 uM) or Akt inhibitor (10 uM) for 1 h. OPG (25 ng/ml) was then added for 90 min. Cells were washed and TRAIL (50 ng/ml) was added for 48 h. Viable colonies were counted after 14 days and data were expressed at fold increase relative to control (untreated) cells. Results are from three independent experiments and express as mean fold increase ± SD. **(F)** CaOV3 cells were preincubated for 1 h with either Akt or ERK1/2 inhibitor and OPG (25 ng/ml) was added for 90 min. Cells were washed and incubated with TRAIL (50 ng/ml) for 24 h, and apoptosis was assessed. Apoptosis is expressed as fold increase relative to control (untreated) cells with the mean of triplicates from three independent experiments ± SD. * *P* < 0.01 compared to TRAIL + OPG treated cells; ** *P* < 0.001.

Because activation of Akt pathway has been closely correlated with TRAIL resistance in ovarian cancer cells [15,26,31] and it is well documented that activation of integrin/FAK signaling may lead to Akt activation [26,29], OPG-mediated activation of Akt was evaluated. The results show that OPG induces a dose-dependent Akt phosphorylation in CaOV3 cells (Figure 3A). OPG induces a rapid phosphorylation of Akt that reaches a peak after 30 min and Akt phosphorylation remained stable for up 120 min (Figure 3B). In concert with these results, OPG treatment of OVCAR3 and OVC238A tumor cells also induces Akt phosphorylation (Figure 3C). Not surprisingly, OPG also induced a dose-dependent activation of ERK in CaOV3 cells (Figure 3D). To further examine the link between OPG-mediated Akt activation and TRAIL attenuation, we used chemical inhibitors to block the activation of the Akt signaling. CaOV3 cells were treated with PI3K inhibitor (LY294002) or specific Akt inhibitor (Akt 1/2 inhibitor) for 1 h followed by addition of OPG. After washing, TRAIL was added and survival was evaluated by clonogenic assay. The inhibition of PI3K/Akt signaling almost completely abrogated the protective effect of OPG (Figure 3E). In contrast, inhibition of ERK1/2 signaling by U0126 had no effect on OPG-mediated protection against TRAIL-induced apoptosis. Consistent with these findings, the inhibition of Akt significantly abrogated OPG-mediated attenuation of TRAIL-induced apoptosis (Figure 3F). All together, these data suggest that Akt signaling is critical for OPG-mediated attenuation of TRAIL-induced apoptosis while ERK signaling does not play a significant role.

### OPG-mediated Akt activation is regulated by integrin/FAK signaling

**Figure 4:**
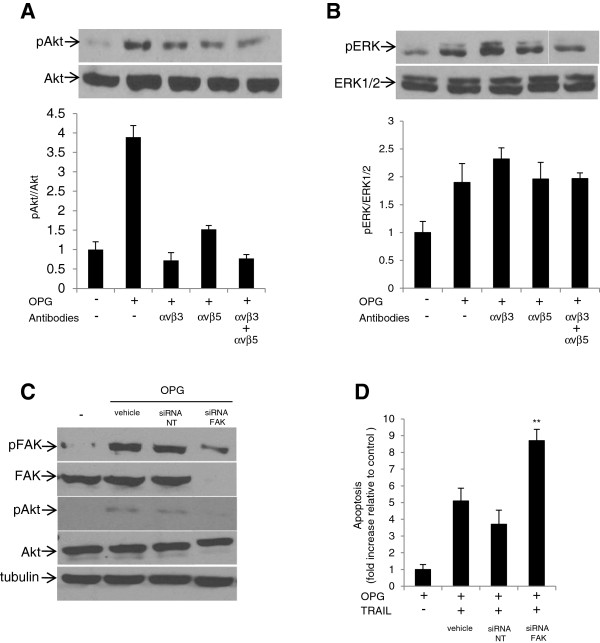
**Integrin/FAK mediates OPG-induced Akt activation.** CaOV3 cells were incubated with αvβ3 and αvβ5 integrin blocking antibodies (5 μg/ml) for 1 h. Cells were washed, incubated with OPG (25 ng/ml) for 90 min and subsequently lysed for immunoblot with **(A)** anti-Akt and anti-phospho-Akt antibodies or **(B)** anti-ERK1/2 or anti-phospho-ERK1/2 antibodies. Densitometric quantification of phosphorylated Akt from three separate experiments normalized to total Akt was done. **(C)** CaOV3 cells were treated with either lipid vehicle alone, non-targeted siRNA (NT siRNA) or FAK siRNA for 24 h. OPG (25 mg/ml) was then added for 90 min and cells were subsequently lysed and immunoblotted for total FAK, phosphorylated FAK, total Akt and phosphorylated Akt. **(D)** CaOV3 cells were preincubated for 24 h with either lipid vehicle alone, non-targeted siRNA (NT siRNA) or FAK siRNA. OPG (25 ng/ml) was then added for 90 min. Cells were washed and incubated with TRAIL (50 ng/ml) for 24 h, and apoptosis was assessed. Apoptosis is expressed as fold increase relative to control (untreated) cells with the mean of triplicates from three independent experiments ± SD. * *P* < 0.01, ** *P* < 0.001 compared to NT siRNA treated cells.

Akt has been described as a downstream signaling mediator for integrin/FAK-mediating event [29]. Akt activation has also been shown to inhibit TRAIL-induced apoptosis in ovarian cancer cells [26,31]. To determine the whether OPG-mediated Akt activation is integrin/FAK-dependent, we examined the effect αvβ3 or αvβ5 blocking antibodies on Akt and ERK1/2 activation in CaOV3 cells. Cells were incubated with anti-integrin blocking antibodies for 1 h, stimulated with OPG for 1 h and cell lysates were assayed by immunoblot for Akt activation. OPG-mediated Akt activation was markedly decreased by αvβ3 or αvβ5 blocking antibodies or a combination of both (Figure 4A). In contrast, OPG-mediated activation of ERK1/2 was unaffected by αvβ3 or αvβ5 blocking antibodies or the combination of both (Figure 4B). To further investigate the role of FAK on OPG-mediated Akt activation, FAK was down-regulated using a FAK siRNA, and Akt activation was assessed by immunoblot. siRNA-mediated down-regulation of FAK strongly inhibited Akt phosphorylation in OPG-stimulated CaOV3 cells (Figure 4C). To further define the contribution of FAK to OPG-mediated attenuation of TRAIL-induced apoptosis, CaOV3 cells were pre-incubated with OPG, washed and treated with TRAIL in the presence of control (NT siRNA) or FAK siRNA (Figure 4D). The down-regulation of FAK expression significantly inhibited the prosurvival effect of OPG. The data suggest that Akt is activated by OPG via αvβ3 or αvβ5 integrins/FAK signaling.

## Discussion

Important aspects of ovarian cancer progression include resistance to drug-induced apoptosis. Early studies have shown that OPG, in paracrine or autocrine manners, functions as a survival factor for tumor cells by preventing apoptosis induced by TRAIL [16-23]. Indeed, soluble secreted OPG has been shown to act as a decoy receptor for TRAIL [11,12]. In addition, OPG has been shown to promote angiogenesis and endothelial cell migration and proliferation by inducing integrin signaling [7-10]. Recent studies have also demonstrated that αvβ5 integrin/Fak signaling attenuates TRAIL-induced apoptosis in ovarian cancer cells by activating Akt survival pathway [26]. These findings prompted us to investigate whether OPG can protect ovarian cancer cells in a TRAIL-binding independent manner. In the present study, we found that OPG attenuates TRAIL-induced apoptosis independently from its binding to TRAIL. Indeed, incubation of ovarian cancer cells with exogenous OPG, followed by removal of OPG and treatment with TRAIL significantly inhibited TRAIL-induced apoptosis (Figure 1), suggesting that OPG may attenuates TRAIL-induced apoptosis via TRAIL binding-dependent and -independent mechanisms.

Previous studies have shown that OPG rapidly activates integrin/FAK signaling in endothelial cells and that OPG-mediated integrin signaling is strongly inhibited by αvβ3 and αvβ5 integrin blocking antibodies [7,8]. Similarly, we showed that OPG activates both αvβ3 and αvβ5 integrin signaling in ovarian cancer cells (Figure 2). These findings suggest that OPG-induced integrin/FAK signaling may be common in all OPG-responsive cell types. In addition, the fact that both OPG and malignant ascites activate integrin/FAK signaling and attenuate TRAIL-induced apoptosis suggest that integrin signaling is central to protect ovarian cancer cells from TRAIL cytotoxicity. Several recent studies have shown that Akt activation is important for ovarian cancer cell survival [15,26,31,32]. In this study, we found that OPG-induced attenuation of TRAIL-induced apoptosis was significantly inhibited by chemical inhibitors of the PI3K/Akt pathway (Figure 3) and that OPG activates Akt in an integrin/FAK-dependent manner in ovarian cancer cells (Figure 4). Furthermore, although ERK1/2 was rapidly activated by OPG, experiments with ERK1/2 inhibitors showed that ERK1/2 activation was not required for OPG-induced attenuation of TRAIL-induced apoptosis (Figure 3).

Akt may be activated by various mechanisms, including growth factor receptors, cytokine receptors and G-protein coupled receptors [33]. However, we found that αvβ3 and αvβ5 integrin blocking antibodies and siRNA-mediated downregulation of FAK almost completely abolish OPG-mediated Akt activation. Therefore, we conclude that integrin/FAK signaling is the main pathway involved in OPG-mediated Akt activation. This is consistent the recent study showing that inhibition of growth factor receptors and G-protein coupled receptors failed to block ascites-induced Akt activation in ovarian cancer cells [26]. The inhibition of αvβ5 integrin/FAK signaling however resulted in the blockade of Akt activation in that study.

In conclusion, we have demonstrated that the αvβ3 and αvβ5 integrin/FAK/Akt pathway is involved in OPG-induced attenuation of TRAIL-induced apoptosis in ovarian cancer cells. Furthermore, the present study provides novel information about the mechanisms by which OPG attenuates TRAIL-induced apoptosis by demonstrating that OPG acts also in a TRAIL binding-independent manner.

## Methods

### Primary tumor cells and cell lines

The study was approved by the institutional review board of the Centre Hospitalier Universitaire de Sherbrooke. Written informed consent was obtained from the patient for the publication of this report and any accompaying images from women that undergone surgery by the gynecologic oncology service for OC. Primary tumor cells isolated from malignant ovarian cancer ascites were supplied by the Banque de tissus et de données of the Réseau de Recherche en Cancer of the Fonds de la Recherche du Québec en Santé (FRQS) affiliated with the Canadian Tumor Repository Network (CTRNet). Primary tumor cells (OVC238A) were isolated as follow: ovarian cancer ascites were centrifuged at 1000 rpm for 15 min and cells were washed twice with OSE medium (Wisent, St-Bruno, Québec, Canada). Cells were then resuspended in OSE medium supplemented with 10% FBS, β-estradiol (10^-8^ M), 2 mM glutamine, antibiotics and fungizone and plated into 75 cm^2^ flasks. All floating cells were removed the next day. Tumor cell samples were used at low passage (< 10). Primary tumor cells (OVC238A) were obtained from patients with advanced serous OC. These cells have been previously described and stained positive for epithelial tumor markers anti-CA125 and cytokeratine 8/18 and negative for fibroblast specific marker fibroblast antigen [24]. The OC cell lines CaOV3 and OVCAR3 were obtained from American Type Culture Collection, (Manassas, VA) and maintained in a humidified 5% CO_2_ incubator at 37°C. Cells were passaged twice weekly. OVCAR3 cells were maintained in RPMI-1640 (Wisent, St-Bruno, QC, Canada) supplemented with 20% FBS, insulin (10 mg/L), glutamine (2 mM) and antibiotics. CaOV3 cells were cultured in DMEM/F12 (Wisent) supplemented with 10% FBS, 2 mM glutamine and antibiotics.

### Reagents

Recombinant human TRAIL was purchased from PeproTech (Rocky Hill, NJ). Recombinant OPG was purchased from R&D Systems (Mineapolis, MN). OPG ELISA was purchased from eBioscience (Vienna, Austria). Antibodies for Akt and FAK were from Cell Signaling. Antibodies for phospho-Akt (Ser-473) and phospho-FAK (Tyr-397) were form Life Technologies (Burlington, ON, Canada). ERK antibody was from Santa Cruz Biotech (Santa Cruz, CA). Integrin-blocking antibodies anti-αvβ3 (clone LM609) and anti- αvβ5 (clone PF16) were from Millipore (Temecula, CA). Anti-tubulin antibody was obtained from Sigma (Oakville, ON, Canada). Akt inhibitor 1/2 (1 L-6-hydroxymethyl-*chiro*-inositol 2(R)-2-*O*-methyl-3-*O*-octadecylcarbonate) was from Calbiochem (San Diego, CA). PI3K inhibitor LY294002 and MEK inhibitor U0126 was purchased from EMD (Billerica, MA).

### Cell viability assays

For clonogenic survival assays, cells were plated into 25 cm^2^ tissue culture plates in standard medium. The next day, cells were incubated for 90 min in medium containing OPG (25 ng/ml). Cells were then extensively washed to remove any OPG and TRAIL (50 ng/ml) was added to fresh medium for 48 h. Cells were then washed with PBS and incubated in fresh medium into 6-well plates at the different densities for 14 days. Cells were fixed and stained with crystal violet. The number of colonies, consisting of > 50 cells, in triplicate was counted.

### Conditioned medium

Once cells have reached confluence, the medium was removed and fresh medium was added. After 48 h, the conditioned medium was removed, centrifuged and stored at -20°C until used.

### Apoptosis

Cells were incubated in medium containing OPG (25 ng/ml) for 1 h. Cells were washed to remove OPG and TRAIL (50 ng/ml) was added to fresh medium for 24 h. The release of nucleosomal DNA into the cytoplasm as a measure of apoptosis was determined using the Cell Death Detection ELISA Kit (Roche, Laval, Québec, Canada) according to the manufacturer’s instruction. The absorbance was determined using a microplate reader at 410 nm.

### siRNA transfection

The FAK and non-targeted (NT) siRNA oligonucleotides were purchased from Dharmacon Research Inc (Ottawa, ON, Canada). Cells were seeded in six-well plates and allowed to adhere for 24 h. Cells (50% confluent) were transfected with a mixture containing Lipofectamine 2000 (Life Technology), OPTIMEM (Life Technology) and siRNA. The siRNA/Lipofectamine complex was then added to the medium. The final concentration of siRNA was 10 mM. Cells were incubated for 4–6 h at 37°C and fresh medium was then added.

### Statistical analysis

Experiments were performed in triplicate, and data presented as mean ± SD. Student’s paired *t*-test was used to analyze differences between the treatment conditions and their controls. The threshold for statistical significance is *P* < 0.05.

## Competing interests

The authors declare that they have no competing interests.

## Authors’ contributions

DL participated in the design of the study and performed most the experiments. IM was responsible for the tissues bank and provided the primary OVC238A tumor cells. CL and PGG provided the clinical samples to the Banque de tissus et de données du Réseau de Recherche en Cancer. CR participated in the design of the study and helped to draft the manuscript. AP conceived the study, participated in its design and drafted the manuscript. All authors read and approved the final version of the manuscript.

## References

[B1] Simonet WS Lacey DL Dunstan CR Kelley M Chang MS Luthy R Nguyen HQ Wooden S Bennett L Boone T Shimamoto G DeRose M Elliott R Colombero A Tan HL Trail G Sullivan J Davy E Bucay N Renshaw-Gegg L Hughes TM Hill D Pattison W Campbell P Sander S Van G Tarpley J Derby P Lee R Boyle WJ Osteoprotegerin: a novel secreted protein involved in the regulation bone density Cell 1997 89 309 319 9108485 10.1016/s0092-8674(00)80209-3

[B2] Yasuda H Shima N Nakagawa N Mochizuki SI Yano K Fujise N Sato Y Goto M Yamaguchi K Kuriyama M Kanno T Murakami A Tsuda E Morinaga T Higashio K Identity of osteoclastogenesis inhibitory factor (OCIF) and osteoprotegerin (OPG): a mechanism by which OPG/OCIF inhibits osteoclastogenesis in vitro Endocrinology 1998 139 1329 1337 9492069 10.1210/endo.139.3.5837

[B3] Dougall WC Molecular pathways: osteoclast-dependent and osteoclast-independent roles of the RANKL/RANK/OPG pathway in tumorigenesis and metastasis Clin Cancer Res 2012 18 326 335 22031096 10.1158/1078-0432.CCR-10-2507

[B4] Vidal K Serrant P Schlosser B van der Broek P Lorget F Donnet-Hughes A Osteoprotegerin production by human intestinal cells: a potential regulator of mucosal immune responses Am J Physiol Gastrointes Liver Physiol 2004 287 G836 G844 10.1152/ajpgi.00428.2003 15521102

[B5] Reid PE Brown NJ Holen I Breast cancer cells stimulate osteoprotegerin (OPG) production by endothelial cells through direct cell contact Mol Cancer 2009 8 49 19604388 10.1186/1476-4598-8-49 PMC2719583

[B6] Malyankart UM Scatena M Suchland KL Yun TJ Clark EA Giachelli CM Osteoprotegerin is an αvβ3-induced, NF-κB-dependent survival factor for endothelial cells J Biol Chem 2000 275 20959 20962 10811631 10.1074/jbc.C000290200

[B7] Kobayashi-Sakamoto M Isogai E Hirose K Chiba I Role of αv integrin in osteoprotegerin-induced endothelial cell migration and proliferation Microvasc Res 2008 76 139 144 18656492 10.1016/j.mvr.2008.06.004

[B8] Kobayashi-Sakamoto M Isogai E Holen I Osteoprotegerin induces cytoskeletal reorganization and activates FAK, Src, and ERK signaling in endothelial cells Eur J Haematol 2010 85 26 35 20331738 10.1111/j.1600-0609.2010.01446.x

[B9] McGonigle JS Giachelli CM Scatena M Osteoprotegerin and RANKL differentially regulate angiogenesis and endothelial cell function Angiogenesis 2009 12 35 46 19105036 10.1007/s10456-008-9127-z PMC12490743

[B10] Benslimane-Ahmim Z Poirier F Delomenie C Lokajczyk A Grelact F Galy-Fauroux I Mohamedi A Fischer AM Heymann D Lutomski D Boisson-Vidal C Mechanistic study of the proangiogenic effect of osteoprotegerin Angiogenesis 2013 16 575 593 23386104 10.1007/s10456-013-9337-x

[B11] Emery JG McDonnell P Burke MB Deen KC Lyn S Silverman C Dul E Appelbaum ER Eichman C DiPrinzio R Dodds RA James IE Rosenberg M Lee JC Young PR Osteoprotegerin is a receptor for the cytotoxic ligand TRAIL J Biol Chem 1998 273 14363 14367 9603945 10.1074/jbc.273.23.14363

[B12] Truneh A Sharma S Silverman C Khandekar S Reddy MP Deen KC McLaughlin MM Srinivasula SM Livi GP Marshall LA Alnemri ES Williams WV Doyle ML Temperature-sensitive differential affinity of TRAIL for its receptors J Biol Chem 2000 275 23319 23325 10770955 10.1074/jbc.M910438199

[B13] Abdulghani J El‒Deiry WS TRAIL receptor signaling and therapeutics Expert Opin Ther Targets 2010 14 1091 1108 20819019 10.1517/14728222.2010.519701

[B14] Goncharenko-Khaider N Lane D Matte I Rancourt C Piché A Targeted ovarian cancer treatment: the TRAILs of resistance Am J Cancer Res 2012 2 75 92 22206047 PMC3236573

[B15] Goncharenko-Khaider N Lane D Matte I Rancourt C Piché A The inhibition of Bid expression by Akt leads to resistance to TRAIL-induced apoptosis in ovarian cancer cells Oncogene 2010 29 5523 5536 20661217 10.1038/onc.2010.288 PMC3007125

[B16] Holen I Croucher PI Hamdy FC Eaton CL Osteoprotegerin (OPG) is a survival factor for human prostate cancer cells Cancer Res 2002 62 1619 1623 11912131

[B17] Corey E Brown LG Kiefer JA Quinn JE Pitts TE Blair JM Vessella RL Osteoprotegerin in prostate cancer bone metastasis Cancer Res 2005 65 1710 1718 15753366 10.1158/0008-5472.CAN-04-2033

[B18] Shipman CM Croucher PI Osteoprotegerin is a soluble decoy receptor for tumor necrosis factor-related apoptosis-inducing ligand/Apo2 ligand and can function as a paracrine survival factor for human myeloma cells Cancer Res 2003 63 912 916 12615702

[B19] Neville-Webbe HL Cross NA Eaton CL Nyambo R Evans CA Coleman RE Holen I Osteoprotegerin (OPG) produced by bone marrow stromal cells protects breast cancer cells from TRAIL-induced apoptosis Breast Cancer Res Treat 2004 86 269 279 15567943 10.1023/b:brea.0000036900.48763.b3

[B20] Holen I Cross SS Neville-Webbe HL Cross NA Balasubramanian SP Croucher PI Evans CA Lippitt JM Coleman RE Eaton CL Osteoprotegerin (OPG) expression by breast cancer cells in vitro and breast tumours in vivo – a role in tumour cell survival? Breast Cancer Res Treat 2005 92 207 215 16155791 10.1007/s10549-005-2419-8

[B21] Fisher JL Thomas-Mudge RJ Elliott J Hards DK Sims NA Slavin J Martin TJ Gillespie MT Osteoprotegerin overexpression by breast cancer cells enhances orthotopic and osseous tumor growth and contrasts with that delivered therapeutically Cancer Res 2006 66 3620 3628 16585187 10.1158/0008-5472.CAN-05-3119

[B22] Rachner TD Benad P Rauner M Goettsch C Singh SK Schoppet M Hofbauer LC Osteoprotegerin production by breast cancer cells is suppressed by dexamethasone and confers resistance against TRAIL-induced apoptosis J Cell Biochem 2009 108 106 116 19544400 10.1002/jcb.22232

[B23] De Toni EN Thieme SE Herbst A Behrens A Stieber P Jung A Blum H Göke B Kolligs FT OPG is regulated by beta-catenin and mediates resistance to TRAIL-induced apoptosis in colon cancer Clin Cancer Res 2008 14 4713 4718 18676739 10.1158/1078-0432.CCR-07-5019

[B24] Lane D Matte I Rancourt C Piché A Osteoprotegerin (OPG) protects ovarian cancer cells from TRAIL-induced apoptosis but does not contribute to malignant ascites-mediated attenuation of TRAIL-induced apoptosis J Ovarian Res 2012 5 34 23153223 10.1186/1757-2215-5-34 PMC3507713

[B25] Cross SS Yang Z Brown NJ Balasubramanian SP Evans CA Woodward JK Neville-Webbe HL Lippitt JM Reed MW Coleman RE Holen I Osteoprotegerin (OPG)-a potential new role in the regulation of endothelial cell phenotype and tumour angiogenesis? Int J Cancer 2006 118 1901 1908 16287088 10.1002/ijc.21606

[B26] Lane D Goncharenko-Khaider N Rancourt C Piché A Ovarian cancer ascites protects from TRAIL-induced cell death through αvβ5 integrin-mediated focal adhesion kinase and Akt activation Oncogene 2010 29 3519 3531 20400979 10.1038/onc.2010.107

[B27] Goncharenko-Khaider N Matte I Lane D Rancourt C Piché A Ovarian cancer ascites increase Mcl-1 expression in tumor cells through ERK1/2-Elk-1 signaling to attenuate TRAIL-induced apoptosis Mol Cancer 2012 11 84 23158473 10.1186/1476-4598-11-84 PMC3526430

[B28] Lane D Cartier A L’Espérance S Côté M Rancourt C Piché A Differential induction of apoptosis by tumor necrosis factor-related apoptosis-inducing ligand (TRAIL) in human ovarian carcinoma cells Gynecol Oncol 2004 93 594 604 15196850 10.1016/j.ygyno.2004.03.029

[B29] Stupack DG Cheresh DA Get a ligand, get a life: integrins, signalling and cell survival J Cell Science 2002 115 3729 3734 12235283 10.1242/jcs.00071

[B30] Cannistra SA Ottensmeier C Niloff J Orta B DiCarlo J Expression and function of β1 and αvβ3 integrins in ovarian cancer Gynecol Oncol 1995 58 216 225 7542622 10.1006/gyno.1995.1214

[B31] Lane D Robert V Grondin R Rancourt C Piché A Malignant ascites protect against TRAIL-induced apoptosis by activating the PI3K/Akt pathway in human ovarian cancer cells Int J Cancer 2007 121 1227 1237 17534891 10.1002/ijc.22840

[B32] Mao Y Xu J Song G Zhang N Yin H Twist2 promotes ovarian cancer cell survival through activation of Akt Oncol Lett 2013 6 169 174 23946798 10.3892/ol.2013.1316 PMC3742652

[B33] Dobbin ZC Landen CN The importance of the PI3K/AKT/mTOR pathway in the progression of ovarian cancer Int J Mol Sci 2013 14 8213 8227 23591839 10.3390/ijms14048213 PMC3645739

